# Effect of Putrescine Treatment on Chilling Injury, Fatty Acid Composition and Antioxidant System in Kiwifruit

**DOI:** 10.1371/journal.pone.0162159

**Published:** 2016-09-08

**Authors:** Qingzhen Yang, Feng Wang, Jingping Rao

**Affiliations:** 1 College of Horticulture, Northwest A&F University, Yangling, Shaanxi Province, 712100, PR China; 2 Department of Life Sciences, Yuncheng University, Yuncheng, Shanxi Province, 044000, PR China; Universidad de Malaga, SPAIN

## Abstract

We investigated the effects of different concentrations (0, 1, 2 and 4 mM) of putrescine on chilling injury, fruit quality, ethylene production rate, fatty acid composition and the antioxidant system of cold-stored kiwifruit (*Actinidia chinensis* Planch. var. chinensis ‘Hongyang’). We achieved a significant decrease in ethylene production, maintained fruit quality and alleviated chilling injury during storage via treatment with 2 mM putrescine. Furthermore, putrescine treatment inhibited increases in superoxide anion production rate and H_2_O_2_ concentration, while maintaining higher membrane lipid unsaturation as well as increased activities of superoxide dismutase and catalase. In addition, putrescine treatment enhanced the activities of antioxidant enzymes related to the ascorbate–glutathione cycle while causing higher levels of ascorbic acid and reduced glutathione. Our results suggest that induced tolerance against chilling injury via putrescine treatment in cold-stored kiwifruit may be due to enhanced antioxidant activity, increased unsaturation of membrane lipids, and inhibited ethylene production.

## Introduction

Kiwifruit, a climacteric fruit, softens and decays rapidly upon harvest [1]. Low temperature effectively delays fruit softening and prolongs the postharvest life of kiwifruits [2]. However, kiwifruit is easy to suffer CI when stored at low but nonfreezing temperatures [3]. CI symptoms in kiwifruit are graining, water-soaking and browning in the skin and flesh, accelerated senescence and susceptibility to decay. These symptoms become more serious after fruit is transferred to room temperature. Although various methods, such as gradual cooling [4], pre-harvest accumulation cold [5], pre-harvest spraying calcium [6] and controlled atmospheres [7], have been confirmed to alleviate CI in kiwifruit, there is still a need for identifying more effective techniques for kiwifruit storage.

Polyamines (PAs), small aliphatic amines with high biological activity, are ubiquitous in vivo [8]. Putrescine (Put), spermine (Spm) and spermidine (Spd) are the major forms of PAs found in plants [9]. Application of exogenous PAs has been reported to inhibite ethylene production and delay fruit ripening and senescence [10, 11]. In addition, a number of studies reported application of exogenous PAs to be effective for increasing resistance to CI in post-harvest horticultural crops, including cucumber [12], zucchini [13], pomegranate [14] and apricot [15]. Therefore, it is possible that CI in horticultural crops could be controlled at a commercial scale using treatments with exogenous PAs. Unfortunately, little information is available on the effect of Put on CI in kiwifruit stored at low temperatures. Moreover, despite it having been proven effective at reducing CI, the mode of action of Put has not been determined. Thus, the purpose of this study was to elucidate the effect of Put on CI, H_2_O_2_ concentration, rate of O_2_^·—^ production, the antioxidant system and fatty acid composition, with the goal to understand better how Put treatments alleviate CI of postharvest kiwifruit during storage at low temperature.

## Materials and Methods

### Plant material and treatments

Kiwifruit (*Actinidia chinensis* Planch. var. *chinensis* ‘Hongyang’) were obtained from a commercial orchard, Zhouzhi, Shaanxi Province, China, where they had been hand-harvested at commercial maturity (soluble solid concentration (SSC): 7.02 ± 0.07%; firmness: 115.55 ± 1.34 N), and then transported for one hour to the postharvest research facilities at Northwest A&F University. Fruit were selected that had a uniform size and were free from visual blemishes and disease, and they were randomly divided into four groups, each with 2100 fruits, which were distributed between three replicates. The four groups were immediately treated as follows: (1) Control: fruit were immersed in distilled water at 20°C (room temperature) for 10 min. (2) 1 mM Put: fruit were immersed in solutions of 1 mM Put for 10 min. (3) 2 mM Put: fruit were immersed in solutions of 2 mM Put for 10 min. (4) 4 mM Put: fruit were immersed in solutions of 4 mM Put for 10 min.

After treatment, the fruit were air-dried via fans at room temperature (20 ± 1°C) for 30 min and stored for up to 90 days at 0°C and 90–95% relative humidity (RH).

Samples were taken, beginning immediately after treatment and following a 15 day interval for evaluation of fruit ethylene production rate, firmness, SSC, and titratable acid (TA). Five fruits per sample were peeled and the pulp tissue cut into slices, mixed and immediately frozen in liquid nitrogen before being stored at –80°C for measurements of the O_2_^·—^ production rate, H_2_O_2_, AsA and GSH concentrations, fatty acid composition and antioxidant enzyme activity. Other samples were removed at 15 day intervals from storage at 0°C and held at 20°C for 5 days to simulate shelf conditions before the CI index and CI incidence were determined. CI incidence was assessed at day 90. We repeated each treatment three times. The experiment was conducted twice and similar results were obtained both times, therefore data from only one of the experiments is presented.

### Chilling injury evaluation

CI index and CI incidence were assessed according to Yang *et al* [16]. as the following formula: CI index (between 0 and 1) = ∑[(CI scale) × (number of fruit at the CI scale)]/(4 × total number of fruit in the treatment).

CI incidence (%) = (number of CI fruit/total number of fruit recorded) × 100.

### Measurement of firmness, SSC, and TA

We measured fruit firmness using a penetrometer (FT327, Effegi, Alfonsine, Italy) with a 7.9 mm diameter probe. Measurements were carried out at two equidistant points on the equatorial axis of each fruit (the peel was removed prior to measurements). The speed of probe was 1 mm s^-1^ and the penetration distance was 5 mm. Firmness was expressed as N. We measured SSC with a digital hand-held refractometer (Atago, Tokyo, Japan) and expressed the results as a percentage on the Brix scale. We determined TA via titration of 20 mL of kiwifruit juice to pH 8.1 with 0.1 M NaOH. TA was expressed as percent citric acid.

### Measurement of ethylene production rate

We measured the ethylene production rate by enclosing fifteen fruit from each treatment in 10 L glass containers for 1.5 h at 0°C. However, measurements at the day of harvest were executed at 20°C. We conducted three independent replications. A 1 mL gas sample of the headspace atmosphere was withdrawn with a syringe. The ethylene production rate was analyzed using a gas chromatograph (GC-14A, Shimadzu, Kyoto, Japan), fitted with a flame ionization detector. Oven, detector, and injector were operated at 100, 120, and 120°C, respectively. Flow rates of the carrier gas (N_2_, H_2_, and air) were 30, 30, and 300 mL min^-1^, respectively. Ethylene production rate was expressed as μLC_2_H_4_·kg^-1^·h^-1^.

### Fatty acid quantification

Total lipids were extracted according to the method of Rui et al [17]. Fatty acids were separated and quantified according to Mirdehghan et al [18] via a gas chromatograph (GC, Hitachi model 663–30) equipped with a flame ionization detector. Identification and quantification of fatty acids were performed via comparison of retention times and peak areas with authentic standards. The ratio of unsaturated/saturated fatty acid was calculated using the formula: (oleic acid + linoleic acid + linolenic acid)/ (palmitic acid + stearic acid).

### Measurements of O_2_^·—^ and H_2_O_2_

We used the method published in Wang and Luo [19] to determine the O_2_^·—^ production rate. The flesh of the fruit (3 g) was ground with 7 mL of 100 mM potassium phosphate buffer (pH 7.8) containing 5% (w/v) polyvinyl polypyrrolidone, 1% (v /v) Triton X-100 and 1.0 mM EDTA at 0°C. The homogenate was centrifuged at 12,000 g and 4°C for 20 min, and the supernatant was used for the determination of the O_2_^·—^ production rate. A standard curve was used to determine the O_2_^·—^ production rate and expressed as μmol·g^–1^ FW·min^–1^.

We used the method published in Patterson *et al*. [20] to determine H_2_O_2_ concentrations. The flesh of the fruit (4 g) was homogenized with 5 mL of acetone (0°C). The homogenate was centrifuged for 15 min at 12,000 g at 4°C, and the supernatant phase was collected to determine the H_2_O_2_ concentration. The H_2_O_2_ concentration was calculated using H_2_O_2_ as a standard (with H_2_O_2_ levels ranging from 10 to 100 μmol) and expressed as μmol·g^–1^ FW.

### Enzyme assays

All enzyme extraction procedures were conducted at 4°C. For SOD and CAT the flesh of the fruit (3 g) was ground with 7 mL of 100 mM potassium phosphate buffer (pH 7.0) containing 1% (v/v) Triton X-100, 5% (w/v) polyvinyl polypyrrolidone and 1.0 mM EDTA. For ascorbate peroxidase (APX), monodehydroascorbate reductase (MDHAR), glutathione reductase (GR) and dehydroascorbate reductase (DHAR) the flesh of the fruit (3 g) was ground with 5 mL of 100 mM sodium phosphate buffer (pH 7.5) containing 1 mM AsA, 0.1 mM EDTA and 2% (w/v) polyvinyl-pyrrolidone. The homogenates were centrifuged at 13,000 g for 20 min at 4°C, and the supernatant was used for the following enzyme assays.

We used the method published in Dhindsa *et al*. [21] to determine SOD activity. The amount of enzyme that could inhibit the reduction of nitro blue tetrazolium (NBT) by 50% was regarded as one unit of SOD activity, which was expressed as U·g^–1^ FW·h^–1^.

We used the method published in Aebi [22] to determine CAT activity. One unit of enzyme activity was defined as the amount that caused a change of 0.01 in the absorbance per minute, and the activity was expressed as U·g^–1^ FW·min^–1^.

We used the method published in Nakano and Asada [23] to determine APX activity. One unit of enzyme activity was defined as the amount that caused a change of 0.01 in the absorbance per minute, and the activity was expressed as U·g^–1^ FW·min^–1^.

We used the method published in Edwards *et al*. [24] to determine GR activity. One unit of enzyme activity was defined as the amount that caused a change of 0.01 in the absorbance per minute, and the activity was expressed as U·g^–1^ FW·min^–1^.

We used the method of Marr`e and Arrigoni [25] to determine MDHAR activity. One unit of enzyme activity was defined as the amount that caused a change of 0.01 in the absorbance per minute, and the activity was expressed as U·g^–1^ FW·min^–1^.

We used the method published in Nakano and Asada [23] to determine DHAR activity. One unit of enzyme activity was defined as the amount that caused a change of 0.01 in the absorbance per minute, and the activity was expressed as U·g^–1^ FW·min^–1^.

### Analysis of AsA, DHA, GSH and GSSG

To determine the concentrations of AsA, dehydroascorbic acid (DHA), GSH and oxidized glutathione (GSSG), the flesh of the fruit (2 g) was ground with 5 mL of ice-cold 5% (w/v) trichloroacetic acid and then the homogenate was centrifuged at 13,000 g for 20 min at 4°C. AsA and Total ascorbate (AsA + DHA) concentrations were detected according to the method of Kampfenkel *et al*. [26]. Then the AsA concentration was subtracted from the total ascorbate amount to determine the amount of DHA present. GSH and GSSG were measured using the method described by Castillo and Greppin [27]. All results were expressed in μmol·g^-1^ FW.

### Statistical analysis

We performed all experiments using a completely randomized design. All statistical tests were carried out using SPSS Version 20.0 (SPSS Inc, Chicago, IL, USA). Data sets were subjected to two-way analysis of variance (ANOVA) with treatment and storage time as factors. We separated means using Tukey-Kramer test. We considered all differences of P < 0.05 as significant. Data are presented as mean ± standard error.

## Results

### Development of CI

**Fig 1:**
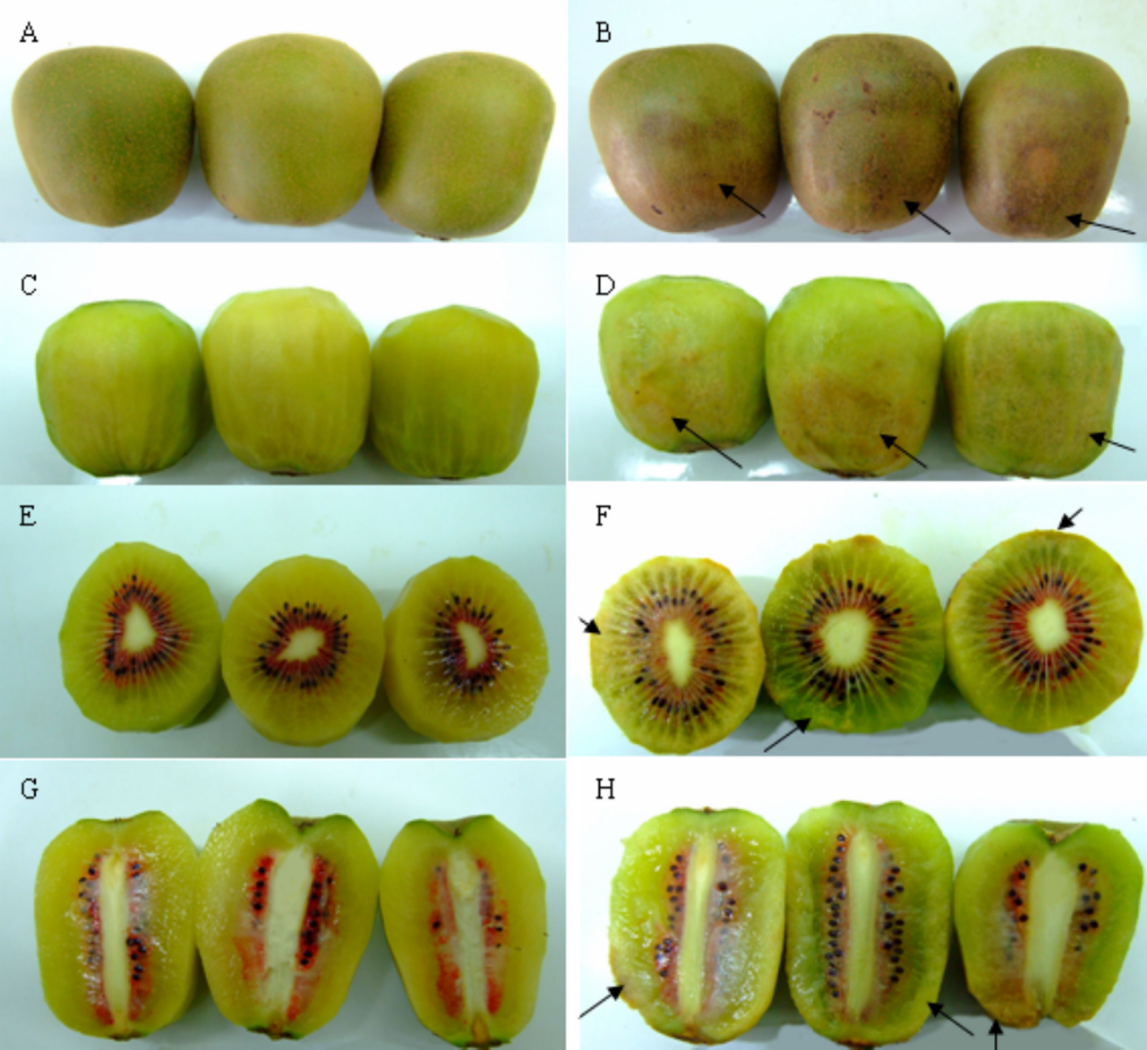
Chilling injury symptoms of ‘Hongyang’ kiwifruit. Representative pictures of ‘Hongyang’ kiwifruit after 90 days of cold storage followed by 5 days of shelf life at 20°C. (A) normal kiwifruit. (B) skin showing the brown symptom of CI (arrow). (C) normal flesh of kiwifruit. (D) flesh of kiwifruit showing the grainy symptom (arrow). (E) the cross-section of the normal flesh. (F) the cross-section of the flesh showing the grainy symptom (arrow). (G)the longitudinal section of the normal flesh. (H) the longitudinal section of the flesh showing the grainy symptom (arrow).

The CI symptoms of ‘Hongyang’ kiwifruit include the flesh becoming grainy and browning, skin browning and increased susceptibility to decay (Fig 1). The graining first appears at the stem end of the outer pericarp and then progresses toward the equator and interior of the fruit, followed by prominent browning and decay of the flesh and skin.

**Fig 2:**
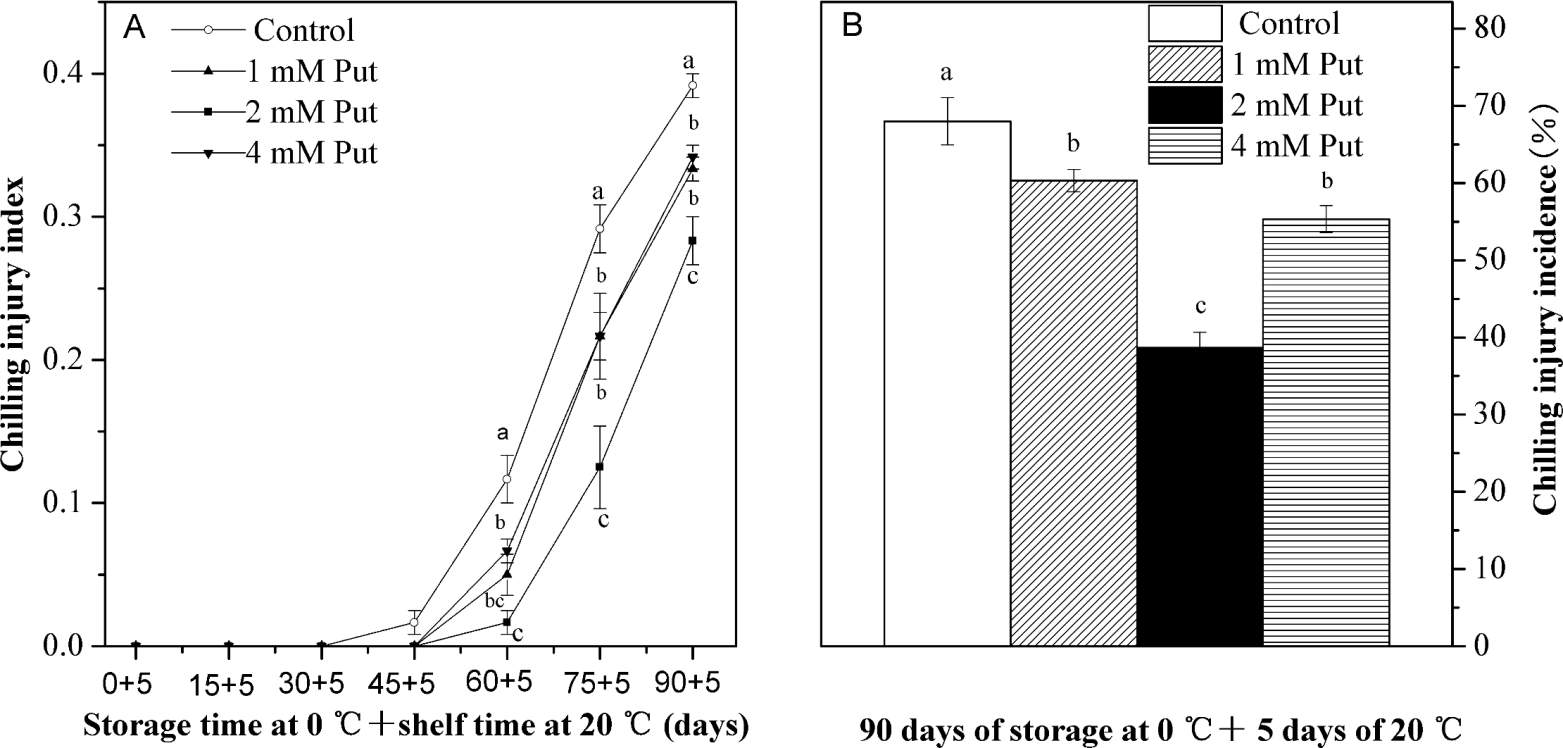
**Effects of exogenous Put treatment on chilling injury index (A) and chilling injury incidence (B) of ‘Hongyang’ fruit.** Kiwifruit were respectively immersed in 0 mM (control), 1 mM, 2 mM and 4 mM putrescine (Put) for 10 min, and then storage at 0°C followed with another 5 days shelf life at 20°C. Chilling injury incidence was assessed at day 90. Vertical bars represent standard error of means (n = 3). Different letters show significant differences between means (*P <0*.*05*).

The control fruit that we stored for 45 d with 5 d shelf life were the first to show CI symptoms, and as we further increased storage time the CI index increased rapidly (Fig 2A). The Put treatments inhibited the increase in CI index, which was significantly lower than in the control fruit between 60 and 90 d storage (*P < 0*.*05*). The Put treatments also significantly inhibited the CI incidence at the end of the 90 d storage (*P < 0*.*05*) (Fig 2B).

We found that treatment with 2 mM Put to be significantly more effective than all other treatments in reducing the CI index development and incidence. At the end of the low temperature storage, the CI index and incidence in 2 mM Put-treated fruit were 28% and 43% lower than those of the control respectively (*P < 0*.*05*).

### Fruit quality parameters

The firmness and TA decreased, while SSC increased in kiwifruit during 0°C storage and subsequent shelf life (Table 1). Put treatment not only resulted in a significantly higher firmness and TA, but also significantly delayed the increase in SSC compared to the control (*P < 0*.*05*). The firmness of fruit treated with 2 mM Put was 33% and 43% higher compared to control fruit at 90 d and subsequent shelf life respectively (*P < 0*.*05*). Levels of SSC in 2 mM Put-treated fruit were 17% and 5% lower compared to control fruit at 90 d and subsequent shelf life respectively (*P < 0*.*05*). In addition this treatment resulted in higher TA concentrations compared to control fruit (*P < 0*.*05*). Therefore, we used the application level of 2 mM Put to determine the attributes of the fruit as shown in the following sections.

**Table 1:** Effects of exogenous Put treatment on firmness, SSC, and titratable acidity of ‘Hongyang’ kiwifruit during storage at 0°C.

Treatment	Storage time (day)	Firmness (N)	SSC (%)	Titratable acidity (%)
	0	116 ± 1.341 aA	7.0 ± 0.074 aC	1.35 ± 0.153 aA
Control	90	23 ± 0.725 cB	15.0 ± 0.178 aA	1.06 ± 0.010 cAB
1 mM Put		29 ± 0.208 bB	14.1 ± 0.171 bB	1.13 ± 0.011 bA
2 mM Put		34 ± 0.416 aB	12.4 ± 0.107 cB	1.23 ± 0.025 aA
4 mM Put		30 ± 1.617 bB	11.8 ± 0.077 cB	1.09 ± 0.013 cAB
Control	90+5	15 ± 0.992 cC	15.1 ± 0.110 aA	0.76 ± 0.031 cB
1 mM Put		22 ± 0.418 bC	15.1 ± 0.061 aA	0.94 ± 0.012 bA
2 mM Put		26 ± 1.077 aC	14.4 ± 0.196 bA	1.11 ± 0.027 aA
4 mM Put		22 ± 0.757 bC	13.3 ± 0.072 cA	0.95 ± 0.010 bB

Kiwifruit were respectively immersed in 0 mM (control), 1 mM, 2 mM and 4 mM putrescine (Put) for 10 min. The storage time is expressed as days of 0°C storage. In storage time, ‘+5’ denotes 5 days of shelf life at 20°C following storage at 0°C. SSC is expressed as soluble solid concentration. Data are shown as the mean ± S.E. Values in the same column with different letters for each day are significantly different at *P <0*.*05*. Lowercase letters represent significant difference among treatment factors, and capital letters represent significant difference among storage time factors.

### Ethylene production rate

**Fig 3:**
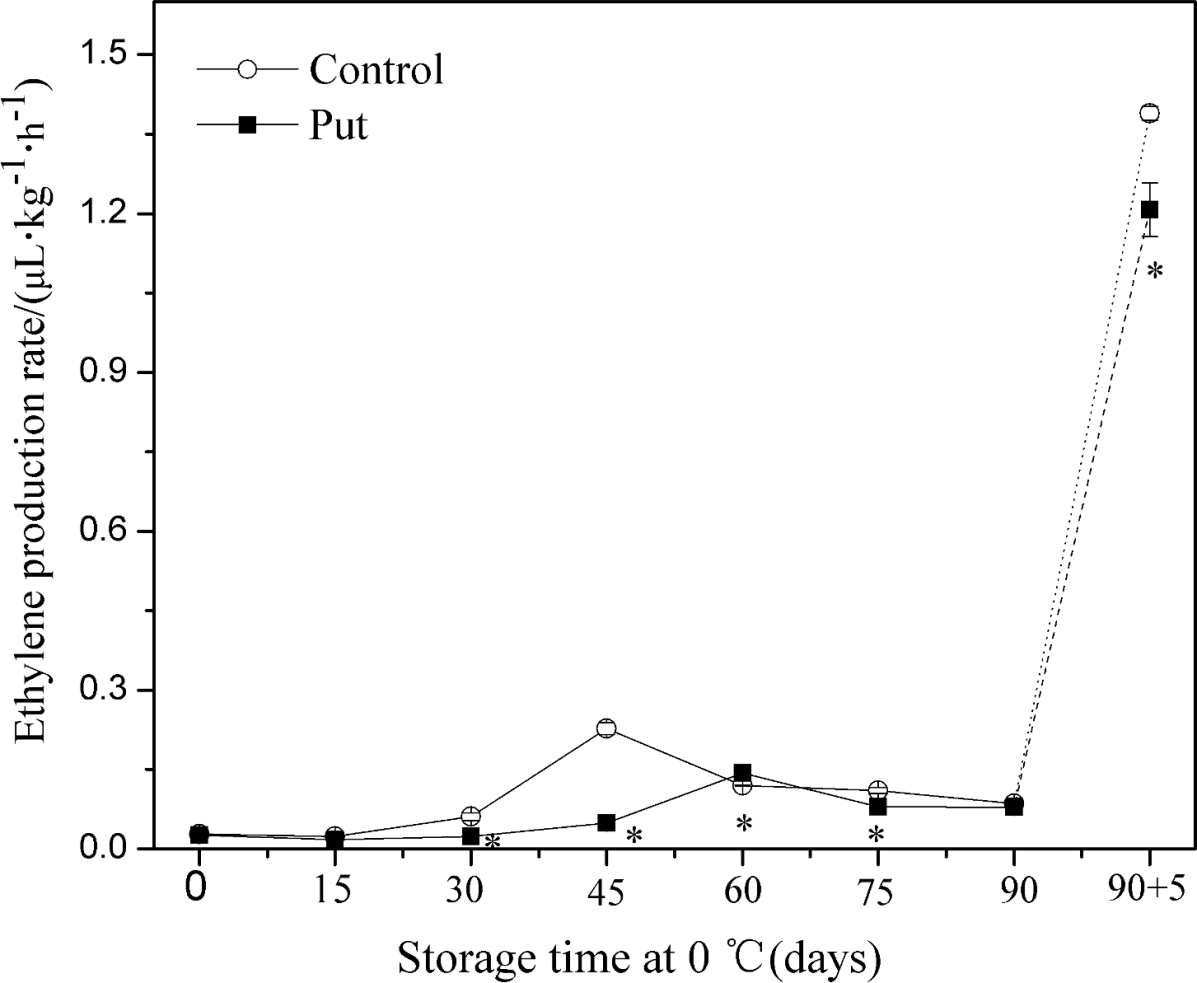
Effect of exogenous Put treatment on ethylene production rate of ‘Hongyang’ fruit. Kiwifruit were respectively immersed in 0 mM (control) and 2 mM putrescine (Put) for 10 min and then storage at 0°C and 90–95% relative humidity for 90 days. In storage time ‘+5’ denotes 5 days of shelf life at 20°C following storage at 0°C. Vertical bars represent standard error of means (n = 3). Asterisks show significant difference (*P <0*.*05*) for the samples between 2 mM Put treatment and control taken at the same time point.

During low temperature storage, the ethylene production rate expressed a typical climacteric pattern (Fig 3). Ethylene production of control fruit increased rapidly to a maximum value at day 45, followed by a rapid decline from day 45 to day 90. After the fruits were transferred to 20°C, ethylene production displayed a further sharp increase. Put not only delayed the expected ethylene climacteric peak by 15 days but reduced the ethylene production rate as well. Ethylene production rate in kiwifruit exposed to Put was on average 56% and 13% lower compared to that in control fruit from 30 to 75 days of storage (except day 60) and after day 90 of storage plus 5 days of shelf life respectively (*P < 0*.*05*).

### Fatty acid composition

**Fig 4:**
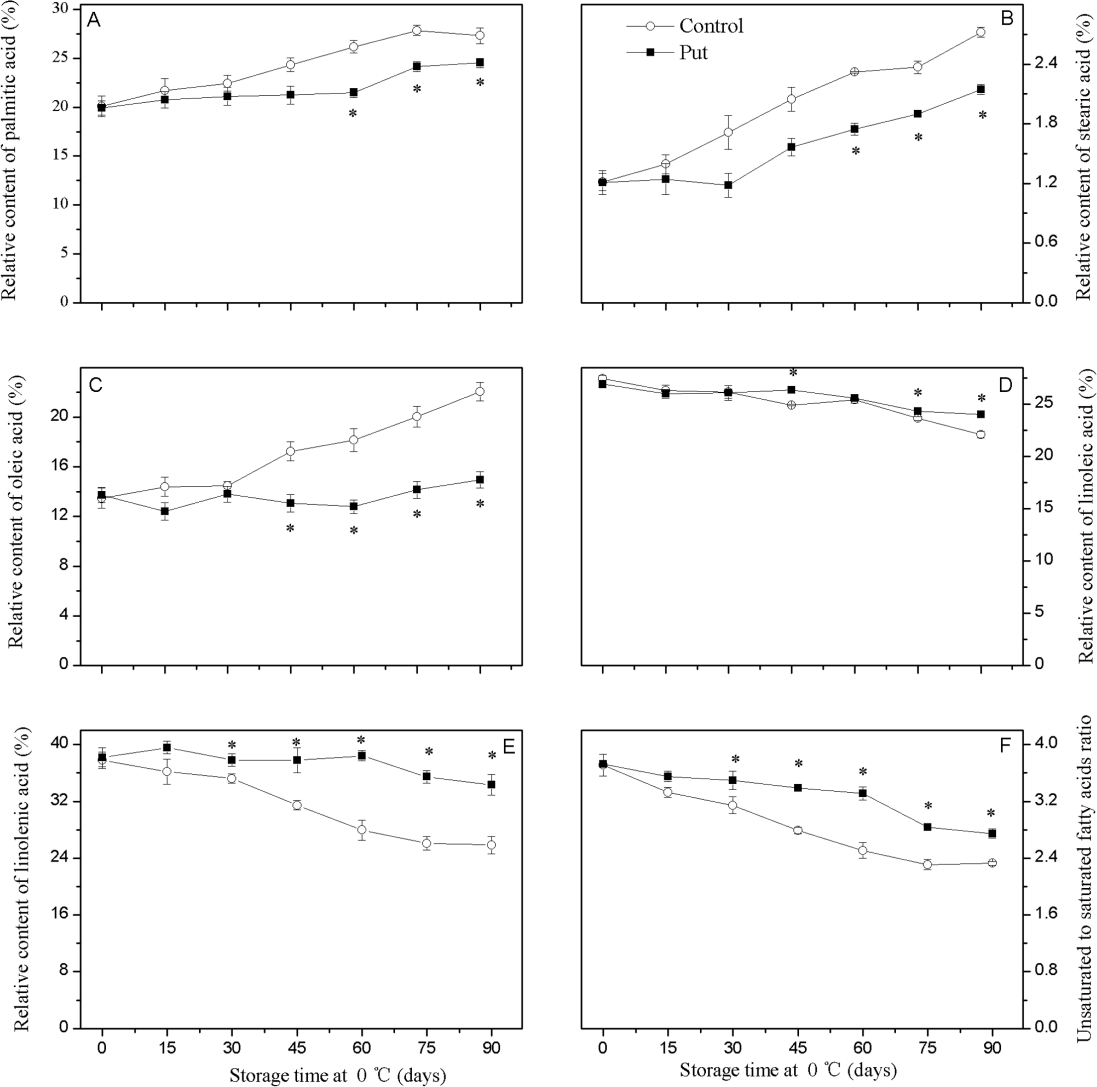
**Effects of exogenous Put treatment on palmitic acid (A), stearic acid (B), Oleic acid (C), linoleic acid (D), linolenic acid concentration (E) and the ratio of unsaturated to saturated fatty acids (F) of ‘Hongyang’ fruit.** Kiwifruit were respectively immersed in 0 mM (control) and 2 mM putrescine (Put) for 10 min and then storage at 0°C and 90–95% relative humidity for 90 days. The ratio of unsaturated to saturated fatty acids denote (oleic acid + linoleic acid + linolenic acid)/ (palmitic acid + stearic acid). Vertical bars represent standard error of means (n = 3). Asterisks show significant difference (*P <0*.*05*) for the samples between 2 mM Put treatment and control taken at the same time point.

Oleic acid, linoleic acid, linolenic acid, stearic acid and palmitic acid were identified and quantified as the major membrane fatty acids of kiwifruit, among which the first three are unsaturated fatty acids and the latter two are saturated fatty acids (Fig 4). During storage, the palmitic and stearic oleic acid concentrations increased gradually. Put treatment inhibited both the increases in palmitic acid and stearic acid, resulting in an average of 14% and 22% lower values compared to control fruit from day 60 to day 90 of storage (*P < 0*.*05*). Throughout storage, the oleic acid concentration increased gradually in control fruits. Put treatment maintained the lower value of oleic acid compared to that of the control fruit. The levels of linoleic acid and linolenic acid decreased gradually during storage. Put treatment inhibited the decrease of these fatty acids. At the end of storage, the amounts of linoleic acid and linolenic acid in Put-treated fruit were 9% and 33%, higher than that of control fruit, respectively. Therefore, Put-treated fruit had a higher unsaturated to saturated fatty acid ratio compared to control fruit during the storage period.

### H_2_O_2_ concentration and O_2_^·—^ production rate

**Fig 5:**
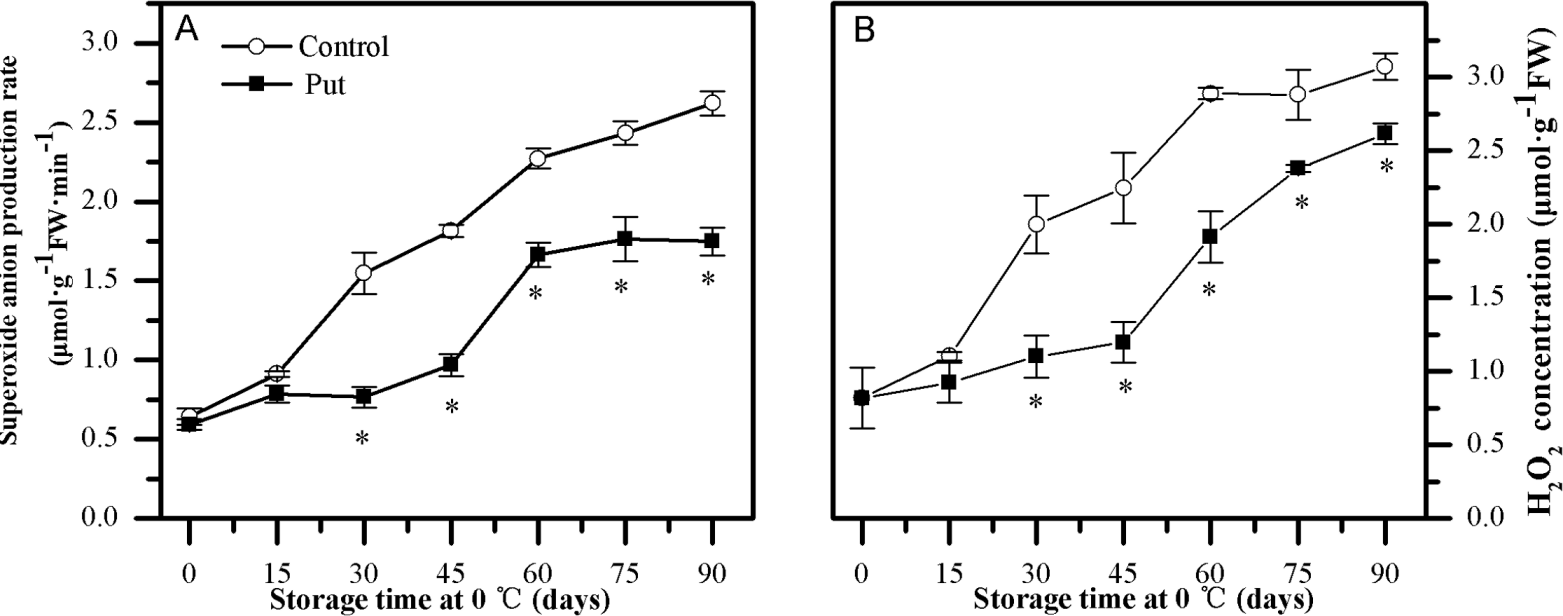
**Effects of exogenous Put treatment on superoxide anion production rate (A) and H_2_O_2_ (B) concentration of ‘Hongyang’ fruit.** Kiwifruit were respectively immersed in 0 mM (control) and 2 mM putrescine (Put) for 10 min and then storage at 0°C and 90–95% relative humidity for 90 days. Vertical bars represent standard error of means (n = 3). Asterisks show significant difference (*P <0*.*05*) for the samples between 2 mM Put treatment and control taken at the same time point.

The levels of O_2_^·—^ and H_2_O_2_ increased slowly and no significant differences were observed between the control and Put treated fruit during the initial 15 days of cold storage (Fig 5). Subsequently, the O_2_^·—^ and H_2_O_2_ concentrations increased rapidly, and the Put treatment significantly inhibited the increases in O_2_^·—^ and H_2_O_2_ (*P < 0*.*05*). The O_2_^·—^ and H_2_O_2_ levels in Put-treated fruit were 37% and 32% lower than those in the control fruit, from day 30 to day 90 of storage respectively (*P < 0*.*05*).

### Activities of SOD and CAT

**Fig 6:**
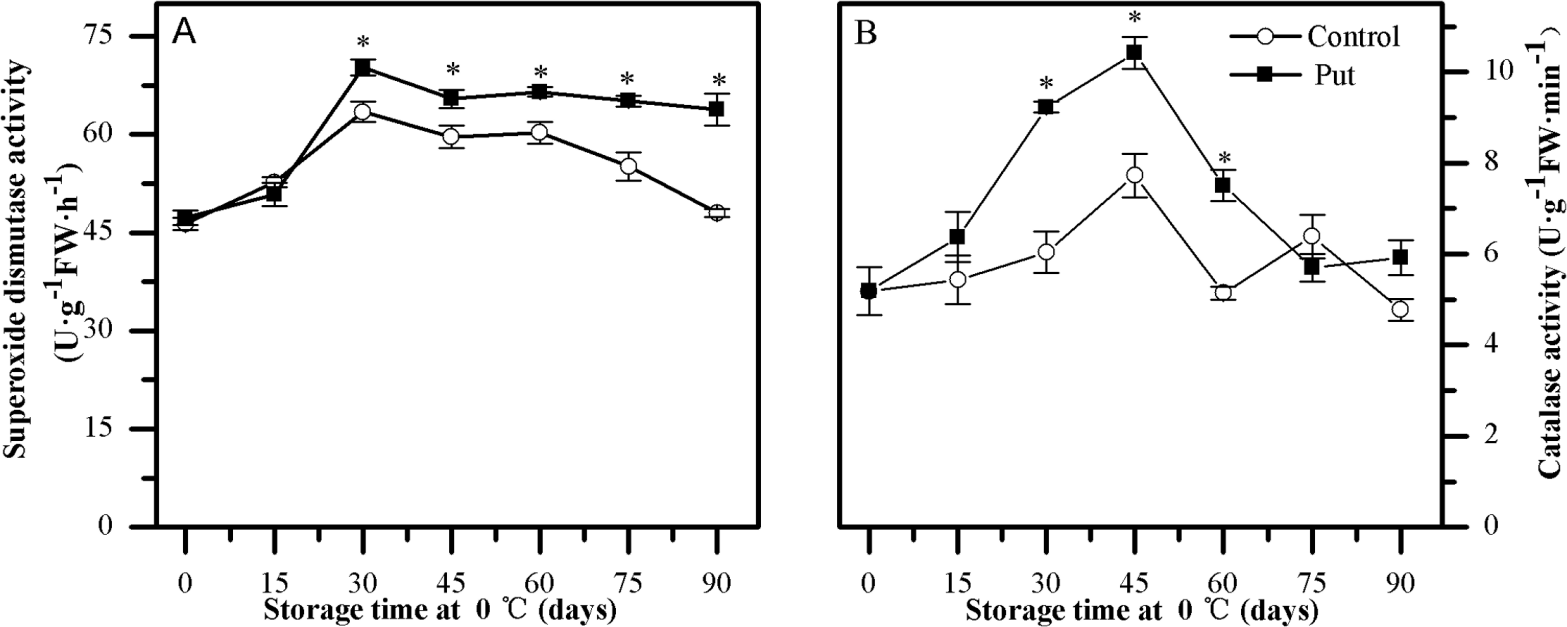
**Effects of exogenous Put treatment on superoxide dismutase (A) and catalase (B) activity of ‘Hongyang’ fruit.** Kiwifruit were respectively immersed in 0 mM (control) and 2 mM putrescine (Put) for 10 min and then storage at 0°C and 90–95% relative humidity for 90 days. Vertical bars represent standard error of means (n = 3). Asterisks show significant difference (*P <0*.*05*) for the samples between 2 mM Put treatment and control taken at the same time point.

The changes in SOD and CAT activities in kiwifruit exhibited similar patterns during cold storage (Fig 6). In control fruit, both enzymes’ activities increased quickly when storage first commenced and then decreased gradually over the remainder of the storage time. The Put treatment significantly promoted the increases and delayed the decreases in the activities of SOD and CAT (*P < 0*.*05*). The activities of SOD and CAT in Put-treated fruit were on average 10% and 45% higher than in control fruit from day 30 to the end of the storage period (except day 75) (*P < 0*.*05*).

### Activities of APX, GR, MDHAR and DHAR

**Fig 7:**
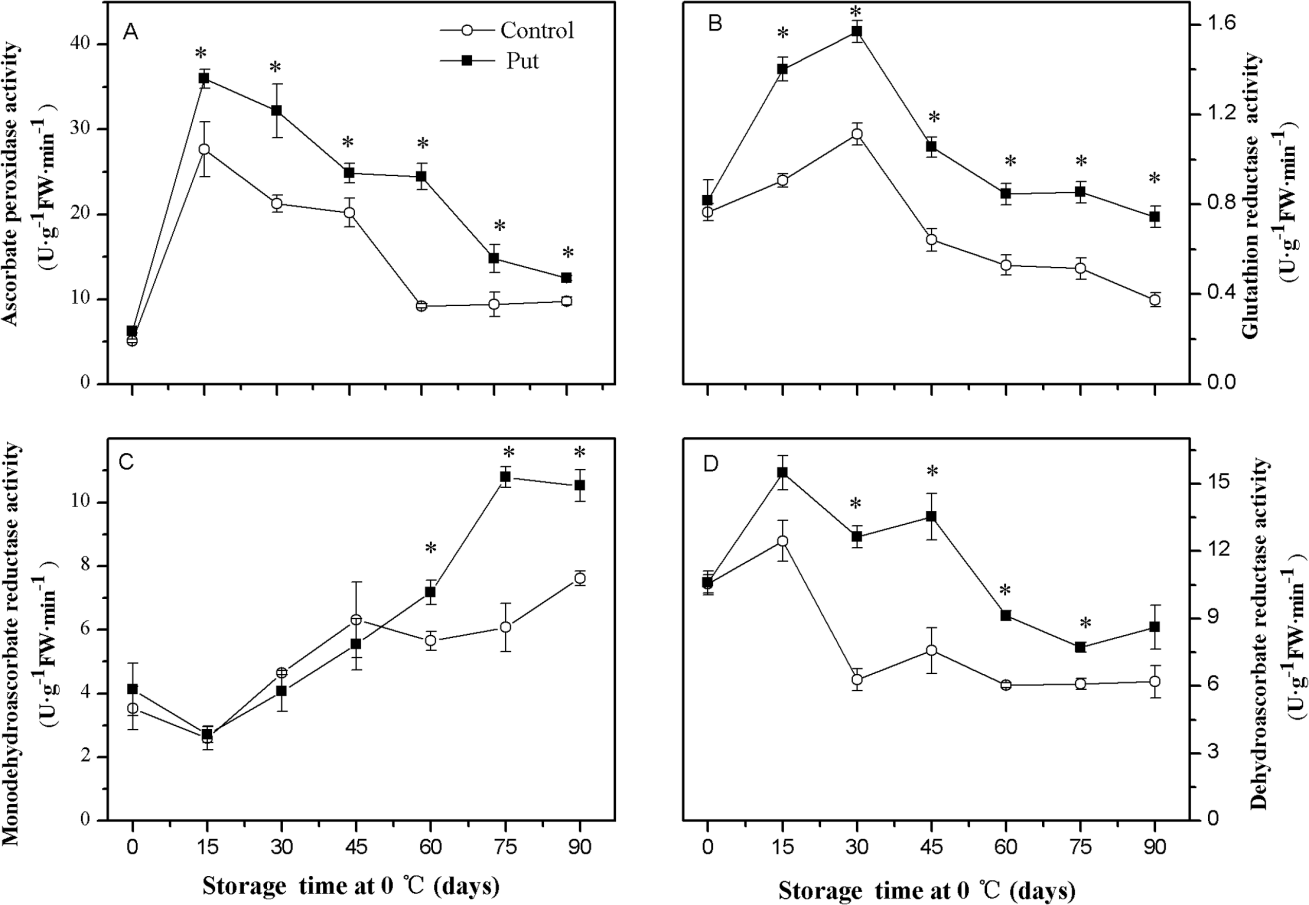
**Effects of exogenous Put treatment on ascorbate peroxidase (A), glutathion reductase (B), monodehydroascorbate reductase (C) and dehydroascorbate reductase (D) activity of ‘Hongyang’ fruit.** Kiwifruit were respectively immersed in 0 mM (control) and 2 mM putrescine (Put) for 10 min and then storage at 0°C and 90–95% relative humidity for 90 days. Vertical bars represent standard error of means (n = 3). Asterisks show significant difference (*P <0*.*05*) for the samples between 2 mM Put treatment and control taken at the same time point.

In the control fruit the activities of APX, GR and DHAR increased quickly when the storage period first started and then decreased gradually toward the end of the period (Fig 7A, 7B and 7D). The Put treatment increased the activities of APX, GR and DHAR and delayed their decreases. The activities of all enzymes were significantly higher in Put treated fruit compared to control fruit during the middle and later storage periods (*P < 0*.*05*).

After an initial short lived decrease, the activities of MDHAR in the control and Put treated fruits increased, but this occurred at a faster rate for the Put treatment in the later part of the storage (Fig 7C). The activities of these enzymes were significantly higher in the Put treated fruit than in the control fruit from day 30 to the end of the storage period (*P < 0*.*05*).

### Concentrations of AsA, DHA, GSH and GSSG

**Fig 8:**
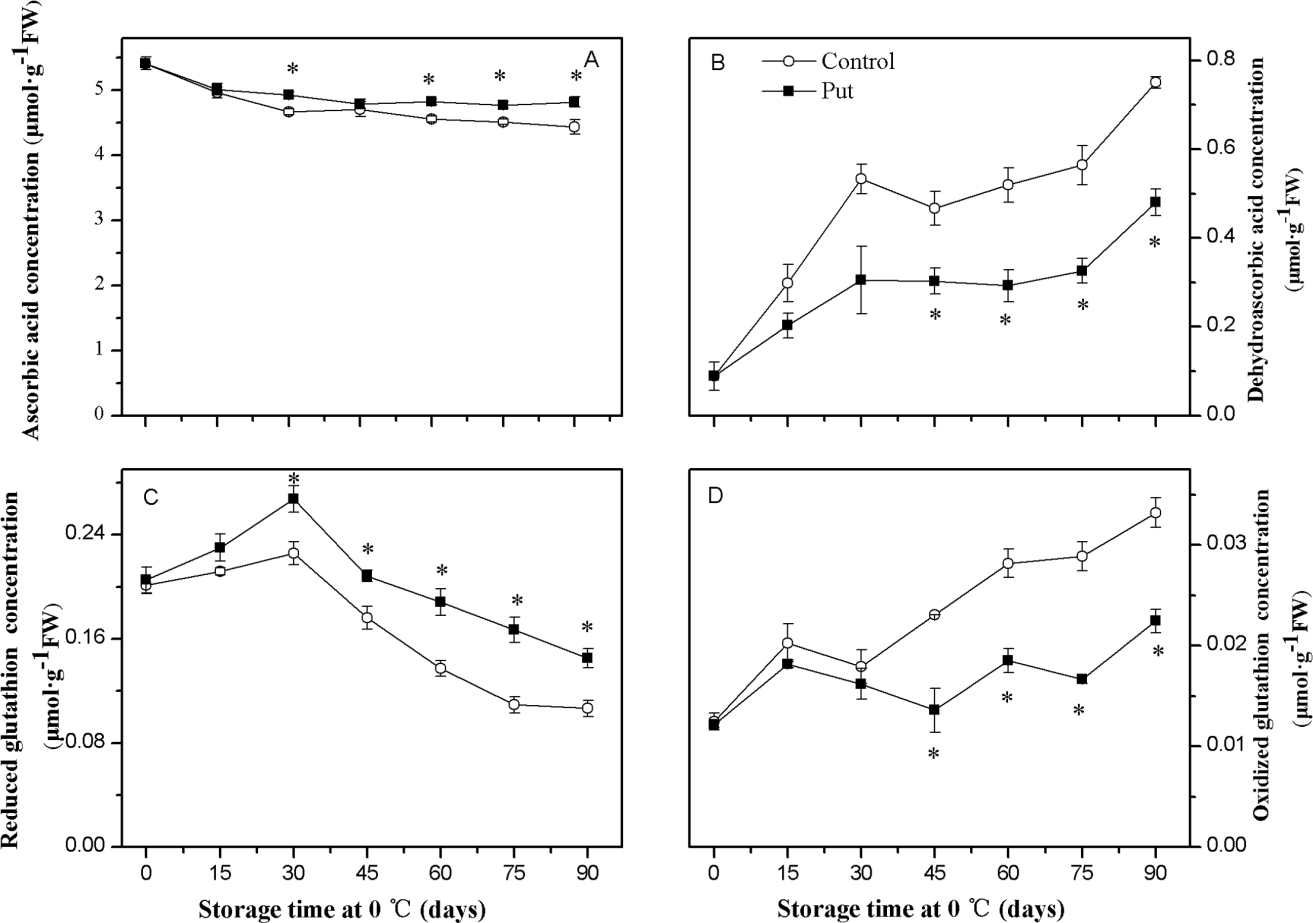
**Effects of exogenous Put treatment on ascorbate acid (A) dehydroascorbic acid (B), reduced glutathione (C), and oxidized glutathione (D) concentration of ‘Hongyang’ fruit.** Kiwifruit were respectively immersed in 0 mM (control) and 2 mM putrescine (Put) for 10 min and then storage at 0°C and 90–95% relative humidity for 90 days. Vertical bars represent standard error of means (n = 3). Asterisks show significant difference (*P <0*.*05*) for the samples between 2 mM Put treatment and control taken at the same time point.

The level of AsA in the control and Put treated fruit decreased gradually during the cold storage, when compared with the accumulation of DHA (Fig 8A and 8B). The Put treatment delayed the decreases in the AsA concentration and inhibited the increases in DHA, which therefore expressed significantly higher AsA levels and lower DHA concentration compared to the control (*P < 0*.*05*).

During the first 30 days of storage the GSH concentrations increased in both the control and Put treated fruit and then decreased (Fig 8C). In contrast, the GSSG concentration increased throughout the storage period (Fig 8D). The Put treatment delayed the decreases in GSH and inhibited the increases in GSSG, which therefore showed a significantly higher GSH level and lower GSSG concentration compared to the control (*P < 0*.*05*).

### PCA analysis

Twenty physical signs of kiwifruit were analyzed by PCA. The results reveal that the accumulative contribution rate of the first three principal components reaches 90%. This indicates that these principal components are expressing information of almost twenty physical signs (Table 2).

**Table 2:** The principal components analysis in kiwifruit (latent value and cumulative contribution).

Principal components	Latent value	Accumulative latent value	Contribution rate /%	Accumulative contribution rate /%
1	12.778	12.778	63.892	63.892
2	3.528	3.528	17.641	81.533
3	1.614	1.614	8.072	89.605
4	1.229	1.229	6.146	95.751

Twenty physical signs of kiwifruit were analyzed by the principal components analysis. These physical signs are oleic acid concentration, linoleic acid concentration, linolenic acid concentration, stearic acid concentration, palmitic acid concentration, (oleic acid + linoleic acid + linolenic acid)/ (palmitic acid + stearic acid), H_2_O_2_ concentration, O_2_^·—^ concentration, superoxide dismutase activity, catalase activity, ascorbate peroxidase activity, glutathione reductase activity, monodehydroascorbate reductase activity, dehydroascorbate reductase activity, dehydroascorbic acid concentration, ascorbic acid concentration, glutathione concentration concentration, oxidized glutathione concentration, C_2_H_4_ production rate and chilling injury index.

We found a positive relationship between the first principal component and H_2_O_2_, MDHAR, saturated fatty acid ratio, stearic acid, palmitic acid and O_2_^·—^ on the direction of the first principal component axis (Table 3). However, there was a negative correlation between the first principal component and the unsaturated to saturated fatty acid ratio, linoleic acid and linolenic acid on the negative side of the first principal component axis. We found a positive relationship between the second principal component and APX, CAT, SOD and DHA in the direction of the second principal component axis. In addition, AsA was negatively correlated to the second principal component, while the same correlation between C_2_H_4_ and the third principal component was detected. Our results indicate that put treatment could alleviate CI by affecting these physical signs.

**Table 3:** Loading of principal components.

		CI	palmitic	stearic	oleic	linoleic	linolenic	Ratio	O_2_^·—^	H_2_O_2_	SOD
Principal components	1	0.898	0.955	0.97	0.693	-0.938	-0.901	-0.971	0.934	0.991	0.462
	2	-0.121	0.104	0.036	-0.272	-0.144	0.105	-0.132	0.128	0.065	0.733
	3	0.365	0.212	-0.143	0.522	-0.164	-0.326	-0.117	-0.293	-0.067	-0.114
		CAT	GR	MDHAR	DHAR	APX	DHA	GSH	GSSG	AsA	C_2_H_4_
Principal components	1	-0.306	-0.642	0.979	-0.806	-0.461	0.812	-0.857	0.668	-0.596	0.644
	2	0.761	0.602	-0.061	0.362	0.818	0.5	0.374	0.311	-0.757	0.116
	3	-0.129	0.401	-0.072	0.241	0.027	0.207	0.193	0.181	0.152	-0.718

Loadings were calculated by the principal components analysis. Palmitic, stearic, oleic, linoleic, linolenic and ratio represent palmitic acid concentration, stearic acid concentration, oleic acid concentration, linoleic acid concentration, linolenic acid concentration and (oleic acid + linoleic acid + linolenic acid)/ (palmitic acid + stearic acid), respectively. SOD, CAT, GR, MDHAR, DHAR, APX, DHA, GSH, GSSG and AsA denote superoxide dismutase activity, catalase activity, glutathione reductase activity, monodehydroascorbate reductase activity, dehydroascorbate reductase activity, ascorbate peroxidase activity, dehydroascorbic acid concentration, glutathione concentration, oxidized glutathione concentration and ascorbic acid concentration, respectively.

## Discussion

Kiwifruit, like many other tropical and subtropical fruit, are easy to suffer CI when stored for prolonged periods at low temperature [3]. It has been reported that PAs could reduce CI and retain fruit quality in pomegranate [14], peach [28] and apricot [15]. Consistent with these reports, Put treatment effectively retains fruit quality (see Table 1) and alleviates CI symptoms in kiwifruit (see Figs 1 and 2), Meanwhile, we also found levels of 2 mM Put to be most effective. These different results could relate to the modification of endogenous polyamines as a result of our treatments. Lower concentration Put might translate into a small amount of endogenous polyamines, which couldn’t fully restrain ROS injury and maintain membrane integrity. While higher concentration Put could lead to excessive accumulation of endogenous polyamine, ultimately causing toxic side effects on plant [29, 30]. However, pinpointing the action mechanism needs further investigation.

PCA analysis revealed the first principal component to be highly correlated to H_2_O_2_, MDHAR, unsaturated to saturated fatty acid ratio, stearic acid, palmitic acid, linoleic acid, O_2_^·—^ and linolenic acid (see Table 3). We found the same strong correlation between the second principal component and APX, CAT, ASA, SOD and DHA, as well as between C_2_H_4_ and the third principal component (see Table 3). Further analysis enabled allocation of these physical signs into these three categories: (1) membrane lipid composition, (2) ROS and antioxidant enzymes and substances, and (3) C_2_H_4_. Our results indicate that these categories will prove useful to understand the underlying mechanism of the CI reducing ability of Put treatment.

It has been suspected that damage of the membrane structure and subsequent changes in lipid constituents is correlated with the occurrence of CI [31]. These changes in lipid composition show mainly decrease in the ratio of unsaturated to saturated fatty acids. This could be affecting the phase transition of membrane lipids from a liquid-crystalline to a solid-gel state, and in so doing lead to membrane peroxidation and damage, accelerating the occurrence of CI. Increasing evidence suggests the benefits of maintaining a higher unsaturated/saturated fatty acid ratio for enhancing fruit tolerance to CI [32, 33]. In this study, we found the kiwifruit of Put treatment displayed higher concentrations of linoleic and linolenic acids and a better ratio of unsaturated to saturated fatty acids than the control group (Fig 4). These results indicate that Put might contribute to maintaining membrane fluidity and alleviating CI in kiwifruit during low temperature storage. A similar effect of Put treatment has been reported for loquat [34] and cucumber [35].

Oxidative stress due to excess production of ROS has been suggested to contribute to the development of CI [1]. Nevertheless, enhancing the activities of antioxidant enzymes and the levels of antioxidant compounds has been shown to contribute to detoxifying ROS and alleviating CI [36–39]. In our study, the increase in H_2_O_2_ concentration and O_2_^·—^ production rate matched the appearance of visual CI parameters during low temperature storage (see Figs 2 and 5). Meanwhile, the higher activities of antioxidant enzymes (SOD, CAT, APX, GR, DHAR and MDHAR) and the levels of antioxidant compounds (GSH and AsA) (Figs 6, 7 and 8) observed in the Put treated fruit may account for the lower levels of O_2_^·—^ and H_2_O_2_ (Fig 5). These results confirm that Put providing protection from CI was related to maintaining higher antioxidant enzymes activity and antioxidant compounds concentration. A similar effectiveness of Put treatment was also obtained in apricot [25].

A decrease in ethylene production is correlated with CI induction [40–41]. Zhang *et al*. report 1-MCP to be sufficient to inhibit ethylene production accompanied with alleviation of CI in persimmon [42]. Through our Put treatment, we were able to suppress the ethylene production rate, effectively leading to alleviated CI (see Figs 2 and 3). Similar effects of treatment with Put have been published for apricot [15] and pomegranate [11].

In conclusion, Put treatment is an effective way to decrease ethylene production, maintain fruit quality, reduce CI, and enhance chilling tolerance. Moderating CI via Put treatment leads to a higher ratio of unsaturated to saturated fatty acids and a lower ethylene production rate. Furthermore, Put induces a more efficient antioxidant system that is beneficial in alleviating oxidative stress and enhancing chilling tolerance of the kiwifruit.

## Supporting Information

S1 Text Data of physiological indicators. Data of chilling injury index, chilling injury incidence, ethylene production rate, palmitic acid concentration, stearic acid concentration, oleic acid concentration, linoleic acid concentration, linolenic acid concentration, the ratio of unsaturated to saturated fatty acids, superoxide anion production rate, H_2_O_2_ concentration, superoxide dismutase activity, catalase activity, ascorbate peroxidase activity, glutathion reductase activity, monodehydroascorbate reductase activity, dehydroascorbate reductase activity, ascorbate acid concentration, dehydroascorbic acid concentration, reduced glutathione concentration, and oxidized glutathione concentration. Data are shown as the mean ± S.E.
(DOC)
