# Supplementary material for: Effect of Putrescine Treatment on Chilling Injury, Fatty Acid Composition and Antioxidant System in Kiwifruit
Source: PLoS One. 2016 Sep 8;11(9):e0162159. doi: 10.1371/journal.pone.0162159 (PMC5015990; doi:10.1371/journal.pone.0162159)
Supplement: S1 Text — Data of chilling injury index, chilling injury incidence, ethylene production rate, palmitic acid concentration, stearic acid concentration, oleic acid concentration, linoleic acid concentration, linolenic acid concentration, the ratio of unsaturated to saturated fatty acids, superoxide anion production rate, H2O2 concentration, superoxide dismutase activity, catalase activity, ascorbate peroxidase activity, glutathion reductase activity, monodehydroascorbate reductase activity, dehydroascorbate reductase activity, ascorbate acid concentration, dehydroascorbic acid concentration, reduced glutathione concentration, and oxidized glutathione concentration. Data are shown as the mean ± S.E. (DOC) [file pone.0162159.s001.doc]

| **Fig. 2 Data of chilling injury index (A)** | | | | | | | | | | | | | |
| --- | --- | --- | --- | --- | --- | --- | --- | --- | --- | --- | --- | --- | --- |
|  | control(%) | | | | 1mM put treatment(%) | | | 2mM put (%) | | | 4mM put (%) | | |
| Storage time at 0 °C | mean | | Stand error | | mean | Stand error | | mean | Stand error | | mean | Stand error | |
| 0 | 0 | | 0 | | 0 | 0 | | 0 | 0 | | 0 | 0 | |
| 15 | 0 | | 0 | | 0 | 0 | | 0 | 0 | | 0 | 0 | |
| 30 | 0 | | 0 | | 0 | 0 | | 0 | 0 | | 0 | 0 | |
| 45 | 0.0167 | | 0.008 | | 0 | 0 | | 0 | 0 | | 0 | 0 | |
| 60 | 0.1167 | | 0.017 | | 0.050 | 0.014 | | 0.017 | 0.008 | | 0.067 | 0.008 | |
| 75 | 0.2917 | | 0.017 | | 0.217 | 0.017 | | 0.125 | 0.029 | | 0.217 | 0.030 | |
| 90 | 0.3917 | | 0.008 | | 0.333 | 0.008 | | 0.283 | 0.017 | | 0.347 | 0.008 | |
|  |  | |  | |  |  | |  |  | |  |  | |
| **Fig. 2 Data of chilling injury ratio (B)** | | | | | | | | | | | | | |
|  | | control(%) | | 1mM put treatment(%) | | | 2mM put (%) | | | | 4mM put (%) | | |
| Storage time at 0 °C | | mean | Stand error | mean | | Stand error | mean | | | Stand error | mean | | Stand error |
| 90 | | 68 | 3.055 | 60.333 | | 1.453 | 38.667 | | | 2.028 | 55.333 | | 1.764 |

| Fig. 3 Data of ethylene production rate | | | | |
| --- | --- | --- | --- | --- |
|  | control(%) | | 2mM put (%) | |
| Storage time at 0 °C | mean | Stand error | mean | Stand error |
| 0 | 0.02801 | 0.00374 | 0.02599 | 0.00424 |
| 15 | 0.02401 | 0.00796 | 0.01706 | 0.00501 |
| 30 | 0.06083 | 0.00733 | 0.02366 | 0.00731 |
| 45 | 0.22722 | 0.01125 | 0.04866 | 0.00655 |
| 60 | 0.11994 | 0.00109 | 0.14372 | 0.00622 |
| 75 | 0.11026 | 0.00574 | 0.07988 | 0.00538 |
| 90 | 0.08557 | 0.01036 | 0.07885 | 0.0099 |
| 90+5 | 1.3894 | 0.01304 | 1.20726 | 0.05088 |
|  |  |  |  |  |
| Fig. 4 Data of palmitic acid (A) | | | | |
|  | control(%) | | 2mM put (%) | |
| Storage time at 0 °C | mean | Stand error | mean | Stand error |
| 0 | 20.09504 | 1.04137 | 19.93975 | 0.73711 |
| 15 | 21.72746 | 1.20185 | 20.74476 | 0.83333 |
| 30 | 22.45902 | 0.78599 | 21.11646 | 0.93333 |
| 45 | 24.33739 | 0.69602 | 21.2478 | 0.92796 |
| 60 | 26.1947 | 0.66583 | 21.47219 | 0.45826 |
| 75 | 27.85354 | 0.52387 | 24.14295 | 0.49329 |
| 90 | 27.31024 | 0.81104 | 24.5363 | 0.46667 |
|  |  |  |  |  |
| Fig. 4 Data of stearic acid (B) | | | | |
|  | control(%) | | 2mM put (%) | |
| Storage time at 0 °C | mean | Stand error | mean | Stand error |
| 0 | 1.21499 | 0.08819 | 1.21093 | 0.1193 |
| 15 | 1.39383 | 0.09452 | 1.24433 | 0.15504 |
| 30 | 1.71332 | 0.16829 | 1.18216 | 0.11921 |
| 45 | 2.0469 | 0.12019 | 1.56661 | 0.0887 |
| 60 | 2.31962 | 0.01 | 1.74719 | 0.0611 |
| 75 | 2.36913 | 0.06333 | 1.90074 | 0.02978 |
| 90 | 2.72157 | 0.05 | 2.14655 | 0.04933 |
|  |  |  |  |  |
| Fig. 4 Data of oleic acid (C) | | | | |
|  | control(%) | | 2mM put (%) | |
| Storage time at 0 °C | mean | Stand error | mean | Stand error |
| 0 | 13.46795 | 0.80829 | 13.71812 | 0.60093 |
| 15 | 14.39246 | 0.76376 | 12.42567 | 0.70238 |
| 30 | 14.47443 | 0.33333 | 13.80927 | 0.63596 |
| 45 | 17.24414 | 0.76376 | 13.08007 | 0.70238 |
| 60 | 18.14582 | 0.93333 | 12.79089 | 0.55478 |
| 75 | 20.05455 | 0.83267 | 14.15023 | 0.67343 |
| 90 | 22.05254 | 0.72648 | 14.94994 | 0.66667 |
|  |  |  |  |  |
| Fig. 4 Data of linoleic acid (D) | | | | |
|  | control(%) | | 2mM put (%) | |
| Storage time at 0 °C | mean | Stand error | mean | Stand error |
| 0 | 27.44362 | 0.33333 | 26.91833 | 0.33333 |
| 15 | 26.28705 | 0.51438 | 25.9848 | 0.4 |
| 30 | 26.1458 | 0.59767 | 26.06939 | 0.68394 |
| 45 | 24.9082 | 0.06667 | 26.34303 | 0.17638 |
| 60 | 25.39509 | 0.29627 | 25.55709 | 0.20817 |
| 75 | 23.62428 | 0.17638 | 24.3261 | 0.18015 |
| 90 | 22.07436 | 0.32648 | 24.0351 | 0.20817 |
|  |  |  |  |  |
| Fig. 4 Data of linolenic acid (E) | | | | |
|  | control(%) | | 2mM put (%) | |
| Storage time at 0 °C | mean | Stand error | mean | Stand error |
| 0 | 37.7784 | 1.1547 | 38.21287 | 1.31318 |
| 15 | 36.19921 | 1.76383 | 39.60043 | 0.88192 |
| 30 | 35.20743 | 0.66667 | 37.82272 | 0.88192 |
| 45 | 31.46337 | 0.66667 | 37.76249 | 1.76383 |
| 60 | 27.94478 | 1.45297 | 38.43264 | 0.72188 |
| 75 | 26.09849 | 0.96667 | 35.47998 | 0.88192 |
| 90 | 25.84129 | 1.24231 | 34.33212 | 1.45297 |
| Fig. 4 Data of The unsaturated/saturated fatty acid ratio (F) | | | | |
|  | control(%) | | 2mM put (%) | |
| Storage time at 0 °C | mean | Stand error | mean | Stand error |
| 0 | 3.70732 | 0.15327 | 3.72934 | 0.02217 |
| 15 | 3.32851 | 0.06781 | 3.5534 | 0.07166 |
| 30 | 3.14646 | 0.12004 | 3.49762 | 0.12926 |
| 45 | 2.79284 | 0.04507 | 3.38731 | 0.0426 |
| 60 | 2.50948 | 0.11239 | 3.31051 | 0.09301 |
| 75 | 2.30948 | 0.07422 | 2.83924 | 0.03295 |
| 90 | 2.32827 | 0.02734 | 2.74701 | 0.06296 |
|  |  |  |  |  |
| Fig. 5 Data of O2·— production rate (A) | | | | |
|  | control(%) | | 2mM put (%) | |
| Storage time at 0 °C | mean | Stand error | mean | Stand error |
| 0 | 0.64239 | 0.05242 | 0.59371 | 0.0337 |
| 15 | 0.91198 | 0.01942 | 0.78513 | 0.05318 |
| 30 | 1.54877 | 0.13057 | 0.76671 | 0.06516 |
| 45 | 1.8155 | 0.03646 | 0.96978 | 0.06947 |
| 60 | 2.2729 | 0.06526 | 1.66473 | 0.07804 |
| 75 | 2.43154 | 0.07386 | 1.7634 | 0.13894 |
| 90 | 2.62056 | 0.0749 | 1.74917 | 0.08786 |
|  |  |  |  |  |
| Fig. 5 Data of H202 concentration (B) | | | | |
|  | control(%) | | 2mM put (%) | |
| Storage time at 0 °C | mean | Stand error | mean | Stand error |
| 0 | 0.81792 | 0.20739 | 0.81792 | 0.20739 |
| 15 | 1.10307 | 0.0271 | 0.92438 | 0.13715 |
| 30 | 1.99818 | 0.19777 | 1.10113 | 0.14358 |
| 45 | 2.24466 | 0.24132 | 1.19723 | 0.13863 |
| 60 | 2.88842 | 0.04077 | 1.91487 | 0.17546 |
| 75 | 2.87936 | 0.16904 | 2.37716 | 0.02433 |
| 90 | 3.07038 | 0.08878 | 2.61565 | 0.07083 |
|  |  |  |  |  |
| Fig. 6 Data of SOD activity (A) | | | | |
|  | control(%) | | 2mM put (%) | |
| Storage time at 0 °C | mean | Stand error | mean | Stand error |
| 0 | 46.36203 | 0.9575 | 47.32305 | 1.11946 |
| 15 | 52.70172 | 0.79155 | 50.81481 | 1.7649 |
| 30 | 63.47223 | 1.57326 | 70.24308 | 1.26261 |
| 45 | 59.64957 | 1.71574 | 65.45347 | 1.36361 |
| 60 | 60.27228 | 1.71444 | 66.53368 | 0.70021 |
| 75 | 55.15214 | 2.15514 | 65.10123 | 0.83414 |
| 90 | 48.04058 | 0.58506 | 63.76963 | 2.44577 |
| Fig. 6 Data of CAT activity (B) | | | | |
|  | control(%) | | 2mM put (%) | |
| Storage time at 0 °C | mean | Stand error | mean | Stand error |
| 0 | 5.18281 | 0.52884 | 5.18281 | 0.52884 |
| 15 | 5.4342 | 0.53357 | 6.37577 | 0.55045 |
| 30 | 6.04075 | 0.44705 | 9.22727 | 0.11996 |
| 45 | 7.72661 | 0.47769 | 10.41723 | 0.35576 |
| 60 | 5.14644 | 0.1435 | 7.50755 | 0.34298 |
| 75 | 6.38453 | 0.47787 | 5.70286 | 0.30197 |
| 90 | 4.77872 | 0.23949 | 5.91866 | 0.3854 |
|  |  |  |  |  |
| Fig. 7 Data of APX activity (A) | | | | |
|  | control(%) | | 2mM put (%) | |
| Storage time at 0 °C | mean | Stand error | mean | Stand error |
| 0 | 5.08811 | 0.18402 | 6.26031 | 0.24951 |
| 15 | 27.63718 | 3.23031 | 35.96603 | 1.1398 |
| 30 | 21.24626 | 0.99826 | 32.19179 | 3.16762 |
| 45 | 20.20337 | 1.66896 | 24.83972 | 1.13617 |
| 60 | 9.20609 | 0.2406 | 24.45252 | 1.55351 |
| 75 | 9.37885 | 1.44372 | 14.77407 | 1.6607 |
| 90 | 9.7829 | 0.44532 | 12.48688 | 0.27768 |
|  |  |  |  |  |
| Fig. 7 Data of GR activity (B) | | | | |
|  | control(%) | | 2mM put (%) | |
| Storage time at 0 °C | mean | Stand error | mean | Stand error |
| 0 | 0.76537 | 0.03722 | 0.81715 | 0.09118 |
| 15 | 0.90667 | 0.03079 | 1.40158 | 0.05223 |
| 30 | 1.11241 | 0.04825 | 1.56832 | 0.04825 |
| 45 | 0.64238 | 0.04999 | 1.05442 | 0.045 |
| 60 | 0.52972 | 0.04521 | 0.84554 | 0.0476 |
| 75 | 0.51458 | 0.04695 | 0.85377 | 0.04806 |
| 90 | 0.3749 | 0.03092 | 0.7442 | 0.04802 |
|  |  |  |  |  |
| Fig. 7 Data of MDHAR activity (C) | | | | |
|  | control(%) | | 2mM put (%) | |
| Storage time at 0 °C | mean | Stand error | mean | Stand error |
| 0 | 3.54109 | 0.67141 | 4.12999 | 0.82932 |
| 15 | 2.58897 | 0.36003 | 2.71555 | 0.25516 |
| 30 | 4.6521 | 0.06862 | 4.07431 | 0.63735 |
| 45 | 6.31279 | 1.18646 | 5.54603 | 0.79733 |
| 60 | 5.65146 | 0.29466 | 7.17452 | 0.39109 |
| 75 | 6.07389 | 0.76314 | 10.79205 | 0.32344 |
| 90 | 7.62016 | 0.23097 | 10.52318 | 0.50018 |
|  |  |  |  |  |
| Fig. 7 Data of DHAR activity (D) | | | | |
|  | control(%) | | 2mM put (%) | |
| Storage time at 0 °C | mean | Stand error | mean | Stand error |
| 0 | 10.54769 | 0.40094 | 10.58924 | 0.52145 |
| 15 | 12.45481 | 0.90421 | 15.49124 | 0.75732 |
| 30 | 6.28336 | 0.48759 | 12.6353 | 0.49738 |
| 45 | 7.57973 | 1.02165 | 13.53199 | 1.03074 |
| 60 | 6.04059 | 0.10127 | 9.12162 | 0.21767 |
| 75 | 6.08091 | 0.24156 | 7.72282 | 0.21683 |
| 90 | 6.19265 | 0.71686 | 8.62026 | 0.9763 |
|  |  |  |  |  |
| **Fig. 8 Data of ASA concentration (A)** | | | | |
|  | control(%) | | 2mM put (%) | |
| Storage time at 0 °C | mean | Stand error | mean | Stand error |
| 0 | 5.41195 | 0.09916 | 5.41195 | 0.09916 |
| 15 | 4.96515 | 0.08078 | 5.01084 | 0.08767 |
| 30 | 4.67002 | 0.04555 | 4.92697 | 0.04275 |
| 45 | 4.70607 | 0.11422 | 4.78733 | 0.07169 |
| 60 | 4.55402 | 0.04608 | 4.82125 | 0.04015 |
| 75 | 4.51438 | 0.04526 | 4.773 | 0.05374 |
| 90 | 4.43672 | 0.10972 | 4.8197 | 0.07818 |
|  |  |  |  |  |
| Fig. 8 Data of DHA concentration (B) | | | | |
|  | control(%) | | 2mM put (%) | |
| Storage time at 0 °C | mean | Stand error | mean | Stand error |
|  | umol•g-1 FW | |  |  |
| Storage time at 0 °C | control(μmol•g-1 FW) | SE | put treatment (μmol•g-1 FW) | SE |
| 0 | 0.08916 | 0.03182 | 0.08916 | 0.03182 |
| 15 | 0.298 | 0.04201 | 0.20281 | 0.02752 |
| 30 | 0.53326 | 0.03328 | 0.30554 | 0.07585 |
| 45 | 0.46685 | 0.03782 | 0.30311 | 0.02957 |
| 60 | 0.51925 | 0.03915 | 0.29248 | 0.03609 |
| 75 | 0.56391 | 0.04411 | 0.32608 | 0.02753 |
| 90 | 0.75006 | 0.01316 | 0.48096 | 0.02991 |
|  |  |  |  |  |
| Fig. 8 Data of GSH concentration (C) | | | | |
|  | control(%) | | 2mM put (%) | |
| Storage time at 0 °C | mean | Stand error | mean | Stand error |
| 0 | 0.20099 | 0.00621 | 0.20526 | 0.00997 |
| 15 | 0.21181 | 0.00284 | 0.23011 | 0.01055 |
| 30 | 0.22561 | 0.00878 | 0.26731 | 0.01006 |
| 45 | 0.17613 | 0.00877 | 0.20821 | 0.00434 |
| 60 | 0.13726 | 0.00611 | 0.18821 | 0.01031 |
| 75 | 0.10937 | 0.00625 | 0.16694 | 0.00985 |
| 90 | 0.10644 | 0.00634 | 0.14497 | 0.0074 |
|  |  |  |  |  |
| Fig. 8 Data of GSSG concentration (D) | | | | |
|  | control(%) | | 2mM put (%) | |
| Storage time at 0 °C | mean | Stand error | mean | Stand error |
| 0 | 0.01247 | 8.54E-04 | 0.01219 | 2.85E-04 |
| 15 | 0.02028 | 0.00188 | 0.01818 | 3.99E-04 |
| 30 | 0.01794 | 0.00166 | 0.0162 | 0.00152 |
| 45 | 0.02309 | 9.29E-18 | 0.01359 | 0.0022 |
| 60 | 0.02819 | 0.0014 | 0.01854 | 0.00121 |
| 75 | 0.02891 | 0.00143 | 0.01664 | 3.72E-04 |
| 90 | 0.03323 | 0.00145 | 0.02247 | 0.00118 |
